# Leading from all levels: building supply chain leadership capacity in Equateur Province, Democratic Republic of Congo

**DOI:** 10.1136/bmjgh-2019-001756

**Published:** 2019-09-03

**Authors:** Nora Phillips-White, Eomba Motomoke, Freddy Nkosi, Jessica Crawford, Bvudzai Priscilla Magadzire, Erin Larsen-Cooper, Franck Biayi

**Affiliations:** 1 Global Technical Team, VillageReach, Seattle, Washington, USA; 2 Democratic Republic of Congo Country Office, VillageReach, Kinshasa, Democratic Republic of Congo; 3 Global Technical Team, VillageReach, Cape Town, South Africa; 4 Programme National d’Approvisionement en Médicaments Essentiels (PNAM), Kinshasa, Democratic Republic of Congo

**Keywords:** immunisation, public health, health systems

## Abstract

A well-functioning supply chain is a critical component of the health system to ensure high-quality medicines and health products are available when and where they are needed. However, because supply chains are complex systems, strong, competent leaders are needed to drive continuous improvement efforts. This paper documents the learnings from a supply chain leadership intervention in the Democratic Republic of Congo (DRC), which aimed to build leadership capacity in a cross-tier group of central/provincial/district-level leaders. The intervention, called the Leadership in Supply Chain Initiative, used an experiential learning curriculum to train 19 ‘champions’ in Equateur Province, DRC. Based on self-assessments and key informant interviews, participants reported that the intervention increased their ability to lead change in the supply chain. In particular, participants and stakeholders noted that empowering district managers as leaders in the supply chain was important to improve supply chain performance, since they oversee service delivery points and are responsible for operationalising changes in the supply chain. Moreover, this intervention adds to evidence that leadership capacity is most effectively gained through experiential learning coupled with mentorship and coaching. Additional research is needed to determine the optimal duration of leadership building interventions and to better understand how supply chain leaders can be supported and mentored within the public health system.

**Summary box**

- Supply chains are an essential component of health systems, and human resources with leadership skills specific to supply chain are needed at all levels of the health system for sustainable, high-performing, responsive and resilient supply chains.
- An experiential learning initiative to build leadership capacity specifically for supply chain improved participants’ perceptions of their abilities to lead change and better support healthcare workers.
- This paper adds to evidence that it is effective to build leadership capacity by combining classroom and experiential learning using an ‘action learning’ approach.
- Additional research is needed to determine the optimal duration of leadership building interventions and to better understand how leaders can be supported and mentored within the public health system.

## Introduction

A well-functioning supply chain is critical in a health system to provide quality health services to the population.^1 2^ Supply chains draw on networks of staff, vehicles, storage and data to procure health commodities from manufactures, transport them to service delivery points and ensure that high-quality products are available at the right time, in the right place and at affordable prices.^1 2^ There is a growing awareness of the importance of strengthening public health supply chains in low/middle-income countries; however, there is a dearth of human resources to provide leadership to drive continuous improvement in these complex systems.^2 3^ Although leadership has been elevated as a priority for health system strengthening in general,^4–12^ there are few frameworks and documented experiences in building capacity to lead public health supply chains.^2 13^

The few programmes working to develop leadership specifically for public health supply chains, such as the Strategic Training Executive Programme (STEP) in East Africa and Lead Lab in India, have focused on the national or provincial level.^13 14^ However, research shows that leadership capacity is also needed at the operational levels of the health system—the district and facility levels—in order to implement and sustain improvements in health services, including supply chain functions.^5 8 12 15 16^ Operational-level personnel often have rich knowledge of on-the-ground obstacles and resources, and are well positioned to take leadership roles in improving supply chain performance.^8 12 15 17^ To address the need for leadership in supply chain at all levels of the health system, the Leadership in Supply Chain Initiative (LSCI) was implemented in Equateur Province, Democratic Republic of Congo (DRC).^18^ This initiative was a collaboration between the Ministry of Health of the DRC and VillageReach, an international non-profit that works with governments to solve healthcare delivery challenges in low-resource communities.^19^

## Setting

The DRC is the largest and most populous Francophone country in Africa, with a population of nearly 77 million people.^20^ Although it is rich in natural resources, the DRC’s poverty rate is among the highest in the world and the country has experienced significant political instability and conflict.^20^ Equateur Province, in the northwest of the country, faces particular challenges in the distribution of health products. The population is dispersed in remote, rural areas divided by the Congo River and its tributaries, which are the major transportation network in the province. Many common supply chain challenges found in low/middle-income countries are exacerbated in Equateur Province, including a limited supply chain workforce, insufficient storage and cold chain equipment, poor quality and insufficient data, and limited transportation infrastructure. As a result, supply chain performance tends to be poor, as indicated by both high stockout rates and high rates of overstock and expiry of health products.^21^

## The Leadership in Supply Chain Initiative

**Figure 1:**
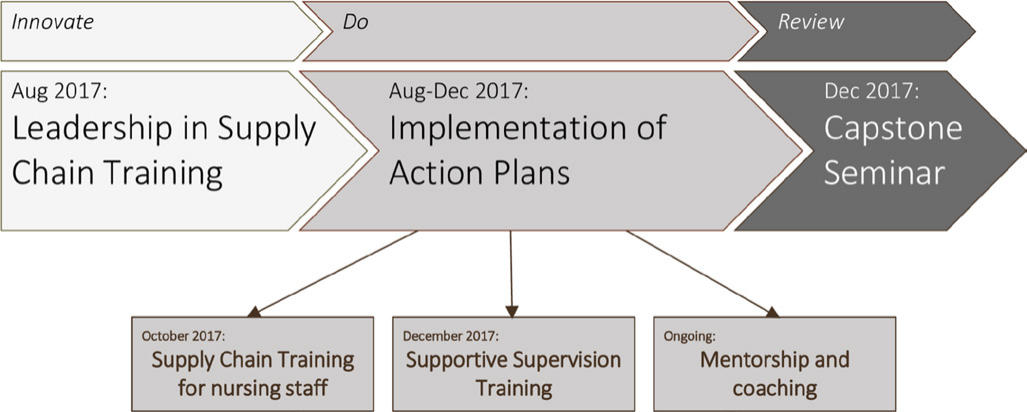
Timeline of the implementation of the Leadership in Supply Chain Initiative in Equateur, Democratic Republic of Congo.

The LSCI was designed to build capacity and position participants, called ‘champions,’ as leaders in the supply chain by engaging them to develop, implement and sustain supply chain improvements. In this way, LSCI used an ‘action learning’ approach, which combines classroom-based training with experiential opportunities to apply skills and has been shown to be a promising approach for building leadership capacity.^6 8–11^ The LSCI integrates action learning with well-known models of change leadership, including Kotter’s 8-step process for leading change^22^ and Lewin’s changing as three steps,^23^ in a three-stage curriculum: ‘Innovate,’ ‘Do’ and ‘Review’ (figure 1). This model was first applied in 2015 through the Lead Lab initiative in India by the USAID|DELIVER project, which focused on leadership capacity at the state level.^13^ The LSCI builds on the work of Lead Lab by extending the intervention to district managers and training national/provincial/district-level champions together to lead change.

## Application of the Leadership in Supply Chain Initiative in Équateur Province, DRC

The LSCI was implemented in Équateur Province, DRC as part of a collaboration between the Ministry of Health and VillageReach. This collaboration, which began in March 2017 and is ongoing, aims to improve the performance of the public health supply chain through a multi-pronged approach. In addition to building supply chain leadership capacity in the health system, it also includes optimising the public health supply chain design and improving the use of data in decision-making.^18^ The subsequent sections of this paper describe the implementation and lessons learnt from the LSCI, which took place from August to December 2017.

### The Innovate stage

In Équateur Province, the LSCI Innovate stage was centred on a 3-day classroom-based workshop in August 2017 that included 19 champions from the central, provincial and district levels. The workshop was facilitated by the VillageReach DRC Country Director, Programme Manager, Supply Chain Advisor and Provincial Project Manager. During the workshop, the facilitators introduced the LSCI model and facilitated classroom-based activities that aimed to provide the champions with foundational change leadership competencies rooted in supply chain. These competencies included team-building, supply chain assessment, advocacy for supply chain as an essential function of the Ministry of Health, mentorship and supportive supervision, and developing and implementing action plans.

As the culmination of the workshop, the champions defined key challenges in the supply chain and created a vision for a short-term change: to improve supply chain management at the district level by December 2017. The champions then developed an action plan for the next 3 months to work towards their vision and practice change leadership. They prioritised two key activities in the action plan: (1) to train frontline healthcare workers in supply chain management, and (2) to improve their own skills in conducting supportive supervision.

### The Do stage

During the Do stage, the champions worked to accomplish the activities they defined in their action plan. Drawing on action learning principles, mentorship and coaching are a key part of the LSCI model, particularly during the Do stage. To this end, VillageReach staff accompanied the champions as they carried out the Do stage. In addition, a WhatsApp group was created with the aim of providing a platform for peer mentoring and for champions to share their experiences, seek and share advice, and build team spirit. The WhatsApp group was moderated by VillageReach staff, who were also available to respond to concerns that the champions could not address themselves, and who occasionally sent messages to check in with the group and initiate discussions after periods of inactivity. The timing of these activities in the Do stage is shown in figure 1.

To train frontline healthcare workers in supply chain management, the provincial and district champions worked together to plan and co-facilitate in-person trainings in three districts for 125 nurses and nurse assistants. These trainings targeted basic supply chain management tasks that nursing staff were responsible for accomplishing in their health centres, including how to inventory products, maintain records, complete order forms and store products safely. Following the trainings, the champions conducted supportive supervision every other month to observe the nursing staff’s supply chain management practices, provide on-the-job training to address gaps or deficiencies and motivate nursing staff by recognising what they were doing well.

The second key activity in the action plan, to improve champions’ skills in conducting supportive supervision, was intertwined with training and support of frontline healthcare workers. VillageReach staff acted as mentors and accompanied supervision teams made up of district and provincial champions to provide on-the-job training in supportive supervision of healthcare workers. Towards the end of the Do stage, the champions decided that it was important to share supportive supervision skills with their peers who were not directly participating in the LSCI. To that end, the champions worked with VillageReach to train 37 provincial and district managers on supportive supervision and on-the-job training in December 2017.

### The Review stage

Finally, in the Review stage, the champions came together for a capstone workshop, which focused on appraising progress on the action plan and continuing to strengthen leadership skills. During the capstone, the champions also developed the next series of actions for supply chain improvement. Based on the experience from the first phase of supply chain improvement, the next series of actions prioritised data collection and use. The capstone also continued to promote teamwork among the champions and across levels to institutionalise the importance of a shared vision and of leading from all levels to achieve the vision together.

## Evaluation

The results of the LSCI were measured through self-evaluations completed by 19 champions as well as through two focus group discussions with nursing staff and open-ended interviews with 8 champions conducted by VillageReach staff following the review phase. Qualitative data were analysed using a general inductive approach to determine thematic codes, which were reviewed and validated by VillageReach’s programme evaluation team.^24^ Results were also presented to Ministry of Health officials involved in the LSCI for review and feedback.

## Lessons learnt

### Effectiveness of action learning in leadership capacity building

Based on the champions’ self-evaluations at the end of the initial workshop (n=19), the vast majority (94%) felt that classroom-based activities in the Innovate stage allowed them to build leadership competencies (table 1). However, at least a third of the champions felt that they could not apply or still needed assistance to apply any given competency. Thus, while the classroom-based activities helped the champions gain some new skills and competencies, they felt they needed additional support to fully master these skills. The LSCI addressed this need by combining classroom-based skills building with experiential opportunities to apply those skills through action learning. This adds to evidence that action learning represents a promising approach for building leadership capacity.^6 8–11^

**Table 1:** Results of the champions' self-assessments

Self-Assessment: Do you believe you have the competency to…	I cannot currently accomplish this	I can accomplish this with assistance	I can accomplish this without assistance
Describe the objectives and concepts of the teamwork approach?	5.56%	33.33%	61.11%
Assess provincial supply chains and determine areas for improvement?	5.56%	55.56%	38.89%
Advocate for supply chains in public health as an essential component of the Ministry of Health?	5.56%	83.33%	11.11%
Support public health workers in acting as ‘change agents’ and ‘champions’ who promote organisational improvements in health service delivery?	5.88%	29.41%	64.71%
Engage a network of leaders in the targeted provinces across health programme?	5.56%	55.56%	38.89%
Engage a network to collaborate with other participants across public/private-sector health supply chains?	5.56%	55.56%	38.89%
Develop a team action plan to improve the provincial supply chain?	5.56%	50.00%	44.44%

### Combining action learning with the resources and opportunities to lead change

In this case, the LSCI was integrated into a larger intervention to improve supply chain performance. This provided the champions with a concrete opportunity to apply and practice their leadership skills in a real-world situation. In doing so, the LSCI built on existing research that leadership is not simply a set of cognitive skills that can be mastered through didactic methods.^6^ Rather, leadership must be cultivated as part of a person’s identity through application and practice in addressing real-world challenges and making changes.^6 8 9 12^ Furthermore, the LSCI seemed to strengthen the overall intervention by helping managers develop leadership skills to drive the changes in supply chain.

In addition, it appears to be important that the overall intervention in Équateur Province was flexible enough to allow the champions to define the actions they wanted to take in the Do stage. Although this intervention did have high-level goals and objectives, it afforded the champions enough space to set their own priorities in their action plans. For example, the supply chain training for nursing staff and supportive supervision training for district and provincial staff were not originally part of VillageReach’s workplan, but there was enough flexibility to provide resources for these activities. Interventions with a leadership capacity building component are unlikely to be effective if they do not provide emerging leaders with genuine opportunities to influence and lead change.

### Building district-level leadership capacity

The LSCI built on supply chain leadership programmes such as Lead Lab^13^ and STEP^14^ by extending this model to the district level. By including champions from the lower levels of the supply chain in defining a vision and plan for change, it helped ensure buy-in from the personnel responsible for day-to-day implementation and oversight. Moreover, it positioned district health managers as champions for making these changes. As one district manager described:

Before (the LSCI), we were a little reluctant (about the changes in the supply chain)…but (the LSCI) changed our way of seeing things. [Before,] everything (all the indicators) was always just poor, poor, poor, poor. But when you have these trainings and you are able to share a vision with the healthcare workers, you start to have hope and optimism about how things can change and become better.

In addition, the focus on leadership specifically for supply chain seemed to be important for the district managers’ ability to lead change. These personnel are typically medical professionals who are well qualified to treat patients, but have had little training in supply chain management or leadership skills and cannot fully leverage their knowledge and experience to improve supply chain performance.^2 3 17^ The focus on supply chain, rather than leadership in general, appeared to be important in increasing district managers’ ability as leaders because they felt more confident in their knowledge of supply chain best practices. As one district manager puts it, “the supervisor needs to know more than the supervisee (to provide effective supervision).” This indicates that interventions aimed at strengthening supply chains should target both technical and leadership skills to empower staff to lead and sustain changes.

### Inclusion of participants across administrative tiers

Several participants noted that a strength of the LSCI was that it brought central/provincial/district-level managers together as a team with the capabilities to support each other in achieving a shared vision. Through work in cross-tier teams, provincial staff were able to observe as district staff built competencies, which seemed to result in increased trust between levels. As a provincial manager noted, “They (district managers) are really beginning to internalise (supply chain leadership competencies).” This supports the existing evidence that cross-tier participation in leadership capacity building is important to ensure that a vision for change is shared across the levels of the health system and helps higher-level officials to recognise leadership capacity at the operational level.^8 11^

In addition, inclusion of both provincial and district staff in field-based activities during the Do stage provided the opportunity for provincial champions to coach district champions in providing supportive supervision for healthcare workers. According to a provincial manager, “Training and capacity building through joint (provincial and district) visits to the (service delivery points)… (allowed for) true capacity building.” As a result, staff at all levels felt supported in developing the necessary skills to lead change in improving supply chain performance.

### Importance of mentoring and supportive supervision

In key informant interviews following the Review stage, champions repeatedly identified on-the-job mentoring and supportive supervision as key factors in improving supply chain performance. For example, many of the nursing staff reported that they had never been formally trained in the logistics functions that they were expected to undertake as part of their jobs such as stock-keeping, ordering and reporting. Participants noted that providing training and coaching for the nursing staff in these skills helped improve stock management in health facilities. During supportive supervision, district-level managers reviewed data management and stock-keeping practices, provided specific feedback for improvement and recognised what workers were doing well. Previously, managers had only told healthcare workers what they were doing wrong, often with limited coaching on how to improve. As one district manager reported:

Many health workers had limited experience in correctly recording and using data. I spent time with them on different data management tools, explaining the importance of each and assisting them to complete each form. At each visit (to) the health centre, I provided them with feedback and recommendations for further improvement.

Furthermore, there was a perception among the champions that building healthcare worker capacity through supportive supervision helped reduce stockouts. A provincial manager explicitly recommended that future efforts continue building on these processes:

[To reduce stockouts,] supportive supervision should be continued and expanded so that more people can master these skills. The more we can support workers at the operational level, the better things will work.

## Limitations

Although the results of the LSCI indicate that it is a promising model for improving supply chain leadership, there are some limitations associated with these results. The evaluation of the LSCI focused on the champions’ perceptions of the LSCI and their observations of changes in supply chain practices and performance. While the qualitative methods used provide important insights about the LSCI, it is not possible to quantify or isolate the effect of the LSCI on the champions’ behaviours or on supply chain indicators. Results may also be subject to social desirability bias due to the relationships between the champions and the VillageReach staff conducting the evaluations.^25^

Furthermore, additional evidence is needed to determine the minimum and optimal durations of leadership training programme with experiential learning components. The LSCI was implemented over 5 months, with 3 months dedicated to the Do stage, while other successful leadership development programme that use action learning lasted for 6–18 months.^8 11 26^ Relatedly, because VillageReach has continued to support supply chain improvement in Équateur as a technical partner of the Ministry of Health, the success of the LSCI in building leadership capacity to sustain high levels of supply chain performance without technical support from an external partner cannot be evaluated. Future investigation should consider how mentoring and coaching for supply chain leaders can be strengthened and sustained within the public health system.

## Conclusion

The LSCI adds to evidence that classroom-based training targeted at individuals is not enough to build capacity to lead improvements in health system performance. Competencies seem to be best learnt when they are applied to address a specific challenge.^6 9–11^ Moreover, integrating the LSCI into supply chain improvement more broadly provided resources to lead changes in a defined time horizon and galvanised the champions to develop a vision of an immediate, achievable future state. This allowed the action learning approach to be effective, because there was genuine opportunity for the champions to practice leadership in improving supply chain performance.

While leadership is often presented as a building block for health systems strengthening,^4^ the LSCI also adds to the evidence that leadership cannot be taught only at the top tiers of the health system. Rather, capable leaders are needed at all levels of the public health supply chain system, as these systems are dynamic and must be responsive to shocks and changes in demand such as outbreaks and natural disasters.^27^ Interventions that aim to build capacity for leadership should create space for leadership and innovation at lower levels of the health system.^6 9 10^ The health of the population depends on having strong leaders with the supply chain skills to cope with complexity and cultivate resiliency and responsiveness.^2 10^
